# Comment on “Effectiveness of Naltrexone in the Prevention of Delayed Respiratory Arrest in Opioid-Naive Methadone-Intoxicated Patients”

**DOI:** 10.1155/2015/752902

**Published:** 2015-02-22

**Authors:** Abbas Aghabiklooei, Hossein Hassanian-Moghaddam, Nasim Zamani

**Affiliations:** ^1^Toxicological Research Center, Loghman-Hakim Hospital, Department of Clinical Toxicology, Faculty of Medicine, Shahid Beheshti University of Medical Sciences, Kamali Avenue, South Kargar Street, Tehran 13336 35445, Iran; ^2^Department of Forensic Medicine, Iran University of Medical Sciences, Tehran 14456 13131, Iran

We read with interest the comments on our recent paper [1, 2]. We think that some points should be clarified to your readers as well as the authors of these comments. Our recent study showed that at least 435 out of 1072 patients (40.5%) in our center were intentionally or accidentally intoxicated by methadone, of whom 216 (20.2%) were younger than 12 years and were admitted to the pediatric ward [3].

Therefore, we believe that the applicability of this therapeutic approach is quite noticeable. We agree that some opioid-naive methadone-intoxicated patients may hide their dependency in history taking but naloxone challenge test will reveal it soon. Starting administration of 0.05–0.1 mg naloxone to each patient even in those who claim not to be dependent is recommended. Withdrawal syndrome caused by oral naltrexone in opioid dependents is usually not life threatening except in ischemic heart disease patients as our previous study showed one death among 132 cases (0.8%) [4]. This fatality is different from long withdrawal precipitated by naltrexone pellet implantation that the authors have mentioned [1]. By the way, we emphasize naloxone challenge test in all patients to prevent withdrawal syndrome.

Although we had no morbidity and mortality in naltrexone group in hospital and in our follow-up clinic, we agree that 50 mg naltrexone may not be enough. The reason why we did not face delayed respiratory depression may be explained by the period of prehospital admission in which methadone half-life had partly passed. This theoretical limitation can be resolved by administration of 150 mg naltrexone to increase its time of effect to 72 hours. Thus, we recommend a naloxone challenge test, giving 50 mg naltrexone if no withdrawal syndrome is observed and giving additional 100 mg naltrexone if the first dose is tolerated.
